# Differential Antimicrobial Efficacy of Preservative-Free Contact Lens Disinfection Systems against Common Ocular Pathogens

**DOI:** 10.1128/spectrum.02138-21

**Published:** 2022-02-09

**Authors:** Rhonda Walters, Allison Campolo, Elise Miller, Cindy McAnally, Manal Gabriel, Paul Shannon, Monica Crary

**Affiliations:** a Alcon Research, LLC, Fort Worth, Texas, USA; Montefiore Medical Center and Albert Einstein College of Medicine

**Keywords:** *Acanthamoeba*, contact lens, contact lens care, disinfection efficacy

## Abstract

Microbial keratitis is a devastating disease that can cause eye damage and blindness and can be the result of infections by several common ocular pathogens. Importantly, some of these pathogens, such as *Acanthamoeba*, are particularly unsusceptible to biocides in common contact lens care solutions. Therefore, the disinfection efficacy of preservative-free (PF) disinfection systems against bacteria, fungi, and *Acanthamoeba* trophozoites and cysts should be assessed as products with the most potential to be efficacious against resistant organisms. PF disinfection systems were analyzed for antimicrobial efficacy. These were the one-step (hydrogen peroxide-based) Clear Care and Clear Care Plus systems and the two-step (povidone-iodine-based) Cleadew system. Stand-alone challenges using bacteria, fungi, and *Acanthamoeba* were prepared according to the International Standards Organization method 14729. These same challenges were also conducted in the presence of the following contact lenses: Boston RGP, Acuvue Oasys, Biofinity, Ultra, and 2-week PremiO. All challenges were performed at the manufacturer’s recommended disinfection time. All preservative-free disinfection systems demonstrated similarly high rates of antimicrobial efficacy when challenged with bacteria or fungi, with or without lenses. However, both Clear Care and Clear Care Plus demonstrated significantly greater disinfection efficacy against *Acanthamoeba* trophozoites and cysts, with and without lenses (*P* < 0.05). Cleadew efficacy was impacted by the addition of contact lenses, whereas Clear Care/Clear Care Plus maintained similar efficacies in the absence or presence of lenses. While both hydrogen peroxide and povidone-iodine are highly effective against bacteria and fungi, hydrogen peroxide maintains significantly greater disinfection capabilities than povidone-iodine against all forms of *Acanthamoeba*.

**IMPORTANCE** Understanding the most efficacious products will allow clinicians to best communicate to patients and consumers the safest products on the market to reduce adverse events, including microbial keratitis, during contact lens use.

## INTRODUCTION

Microbial keratitis is an infectious corneal disease that can lead to irreversible damage to the cornea and permanent vision loss in the absence of prompt diagnosis and treatment (1, 2). The most common form, bacterial keratitis, is most often attributed to Pseudomonas spp., but a range of microorganisms, including fungi, protozoa, and viruses, have also been implicated (3, 4). Risk factors for microbial keratitis vary by geographical location, but the major risk factor in Westernized countries is ineffective contact lens care (5, 6). Lens-related microbial keratitis is typically associated with noncompliant contact lens hygiene practices (7), which may lead to microbial contamination of the contact lenses, storage cases, and storage solutions.

The two main contact lens disinfection systems currently in use are multipurpose solution (MPS) systems and preservative-free disinfection systems which rely on the action of biocides such as hydrogen peroxide (H_2_O_2_) or povidone-iodine. MPS systems are more common and employ disinfectants such as polyhexamethylene biguanide (PHMB; ∼0.0001%), polyquaternium-1 (PQ-1; 0.0001% to 0.001%), myristamidopropyl dimethylamine (MAPD; 0.0005% to 0.0006%), and alexidine (0.0001% to 0.0002%) (8). In contrast, the biocidal activity of hydrogen peroxide systems is predicated on the production of hydroxyl free radicals that attack and penetrate cell membrane lipids and subsequently destroy essential cell components (9). Povidone-iodine has been used as a surgical disinfectant for decades, exerting a biocidal effect by interacting with cell membrane proteins and mitochondrial enzymes (10). Moreover, evidence suggests that preservative-free disinfection systems may potentially provide greater efficacy against bacterial biofilms and *Acanthamoeba* trophozoites and cysts (11–16). Although *Acanthamoeba* keratitis is rare, contact lens wearers account for 85% to 90% of reported cases (17). The increasing incidence and high morbidity (7, 17) of *Acanthamoeba* keratitis necessitate that contact lens disinfecting solutions demonstrate biocidal activity against *Acanthamoeba* trophozoites as well as the persistent and resistant cysts. However, there are currently no international guidelines or standardized procedures for evaluating the biocidal efficacy of contact lens solutions against *Acanthamoeba*.

Clear Care 3% H_2_O_2_ cleaning and disinfecting solution is formulated for the cleaning, disinfection, and storage of soft (hydrophilic) hydrogel, silicone hydrogel, and gas-permeable contact lenses. Clear Care Plus 3% H_2_O_2_ cleaning and disinfecting solution is the more recent version of Clear Care and is formulated with an additional wetting agent to enhance lens surface wettability. Conversely, Cleadew is a povidone-iodine-based cleaning and disinfection system for soft hydrogel, silicone hydrogel, and rigid gas-permeable contact lenses (14). This study compares the antimicrobial efficacies of Clear Care, Clear Care Plus, and Cleadew against bacteria, yeast, mold, and *Acanthamoeba* (cysts and trophozoites) with five different contact lenses.

## RESULTS

**FIG 1:**
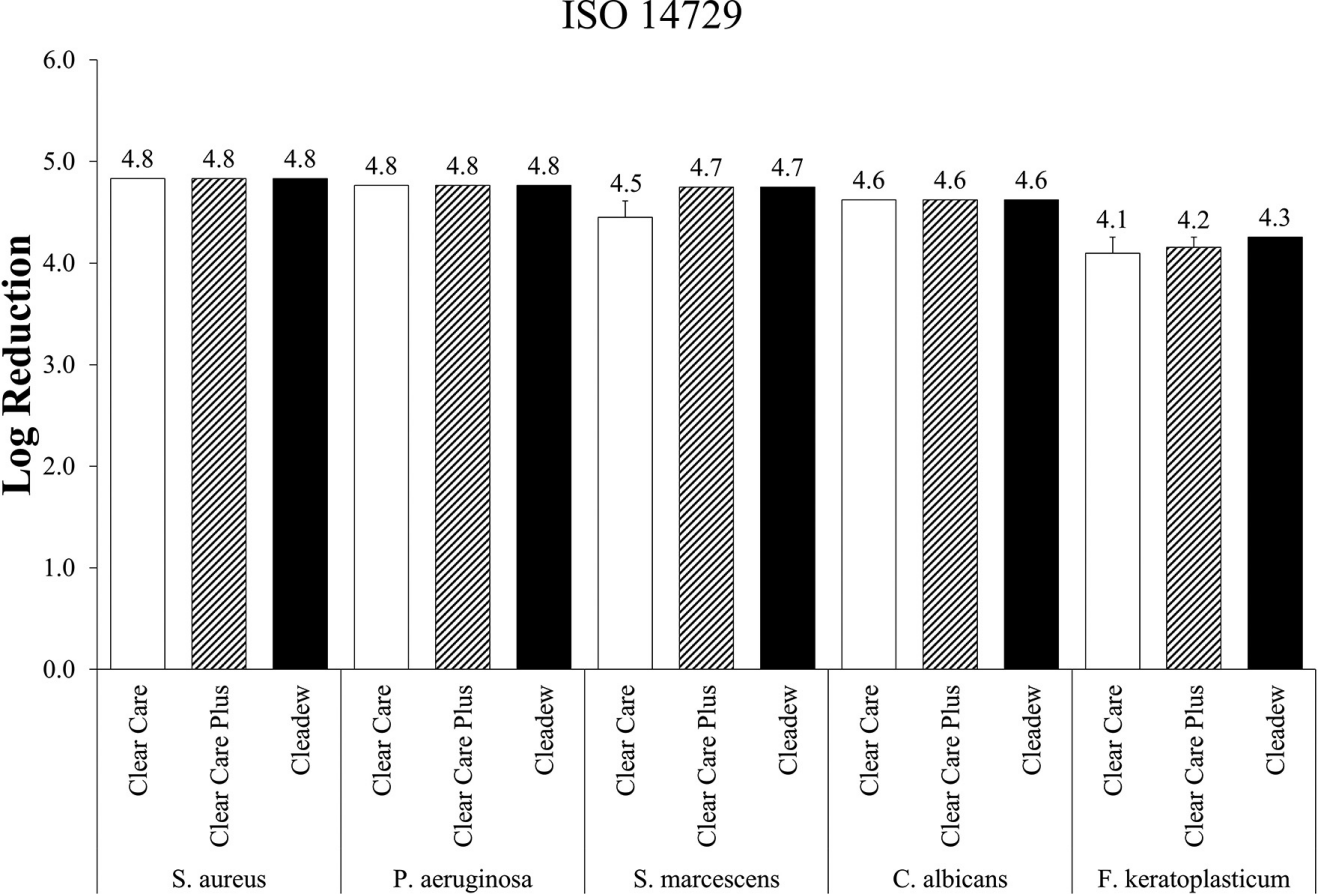
Stand-alone (no-lens) disinfection efficacy of preservative-free disinfection systems against the International Standards Organization (ISO) 14729 organisms, at the manufacturer’s disinfection time. Disinfection efficacy is given as the mean ± standard error log reduction compared to the inoculum control. *n* = 3 to 6/group.

The ISO 14729 organisms (16) Staphylococcus aureus, Pseudomonas aeruginosa, Serratia marcescens, Candida albicans, and Fusarium keratoplasticum were used to evaluate Clear Care, Clear Care Plus, and Cleadew for stand-alone (no-lens) disinfection efficacy (Fig. 1). All products tested met and exceeded the primary criteria for this test for all microorganisms tested, demonstrating greater than a 4 log reduction. There were no differences between products with any of the ISO microorganisms.

**FIG 2:**
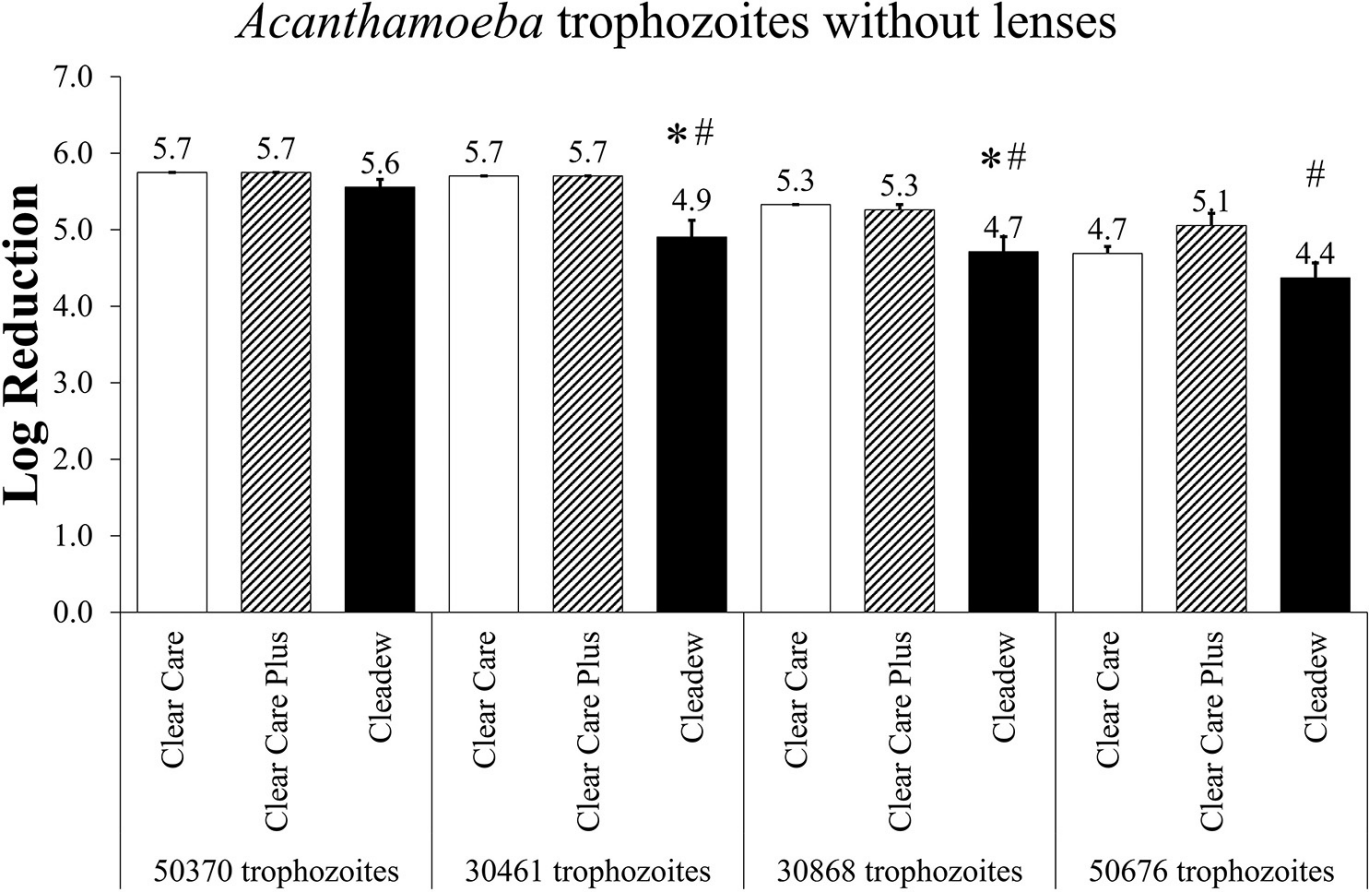
Disinfection efficacy of preservative-free disinfection systems against *Acanthamoeba* trophozoites, at the manufacturer’s disinfection time. Disinfection efficacy is give as the mean ± standard error log reduction compared to the inoculum control. *, *P* < 0.05 versus Clear Care; #, *P* < 0.05 versus Clear Care Plus (one-way ANOVA; *n* = 3 to 6/group).

*Acanthamoeba* trophozoites were subsequently used to evaluate these same products, without lenses (Fig. 2). No difference in efficacy was observed between products with the ATCC 50370 *Acanthamoeba* trophozoites. However, with the ATCC 30461, ATCC 30868, and ATCC 50676 trophozoites, Cleadew demonstrated significantly less disinfection efficacy than Clear Care Plus (*P* < 0.05). Similarly, with the ATCC 30461 and ATCC 30868 strains, Cleadew demonstrated significantly less disinfection efficacy than Clear Care (*P* < 0.05). There were no significant differences between Clear Care and Clear Care Plus.

**FIG 3:**
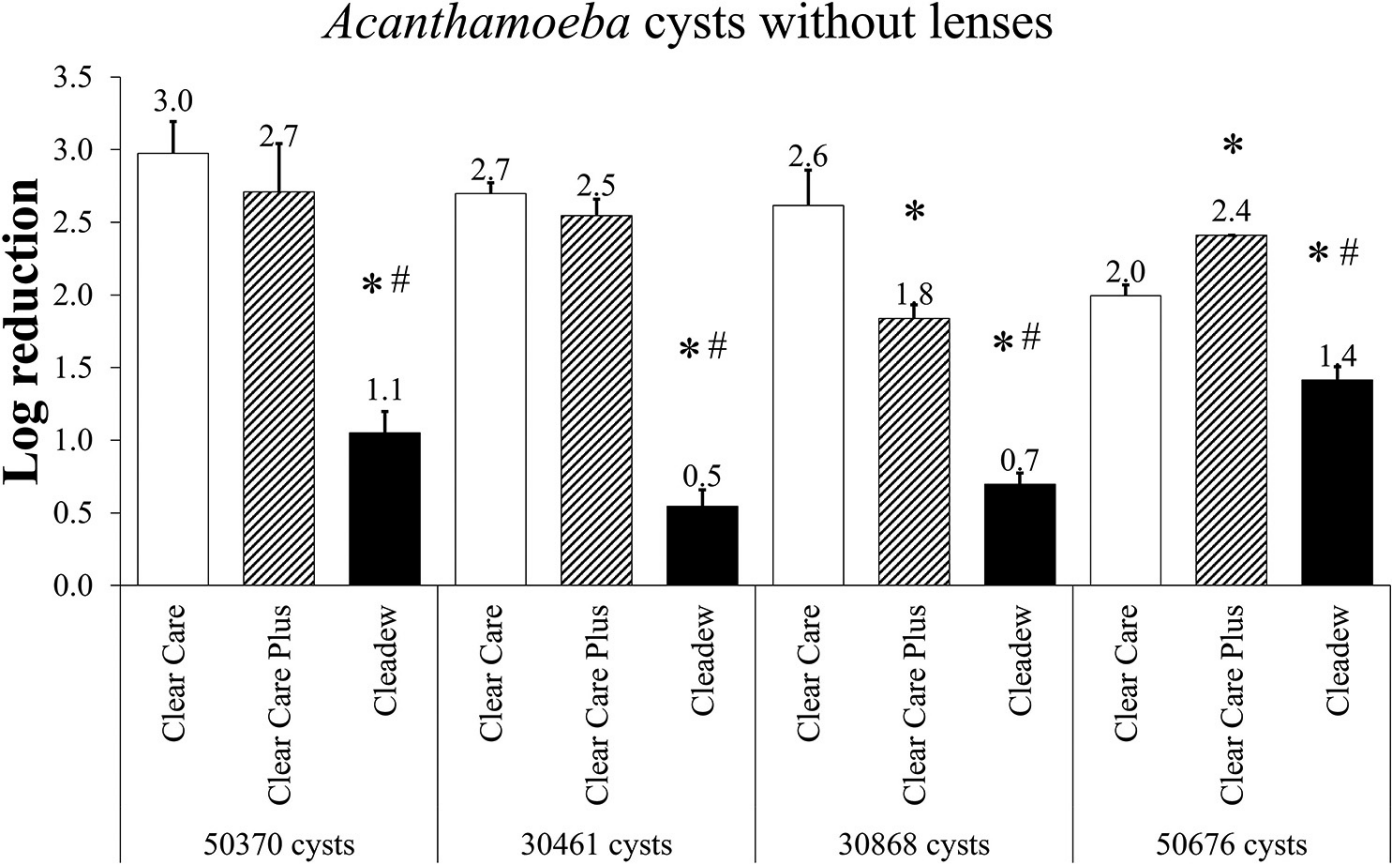
Disinfection efficacy of preservative-free disinfection systems against the *Acanthamoeba* cysts, at the manufacturer’s disinfection time. Disinfection efficacy is given as the mean ± standard error log reduction compared to the inoculum control. *, *P* < 0.05 versus Clear Care; #, *P* < 0.05 versus Clear Care Plus (one-way ANOVA; *n* = 3 to 6/group).

*Acanthamoeba* cysts were also used to evaluate these products, without lenses (Fig. 3). The disinfection efficacy of any product against *Acanthamoeba* cysts is a highly differentiating metric, as cysts are resistant to most biocides. Cleadew demonstrated significantly less disinfection efficacy than Clear Care and Clear Care Plus against ATCC 50370, ATCC 30461, ATCC 30868, and ATCC 50676 *Acanthamoeba* cysts (*P* < 0.05). There were no differences between Clear Care and Clear Care Plus with the ATCC 50370 and ATCC 30461 species. Therefore, it is evident that the hydrogen peroxide-based systems were more efficacious against *Acanthamoeba* cysts than the povidone-iodine-based system.

**FIG 4:**
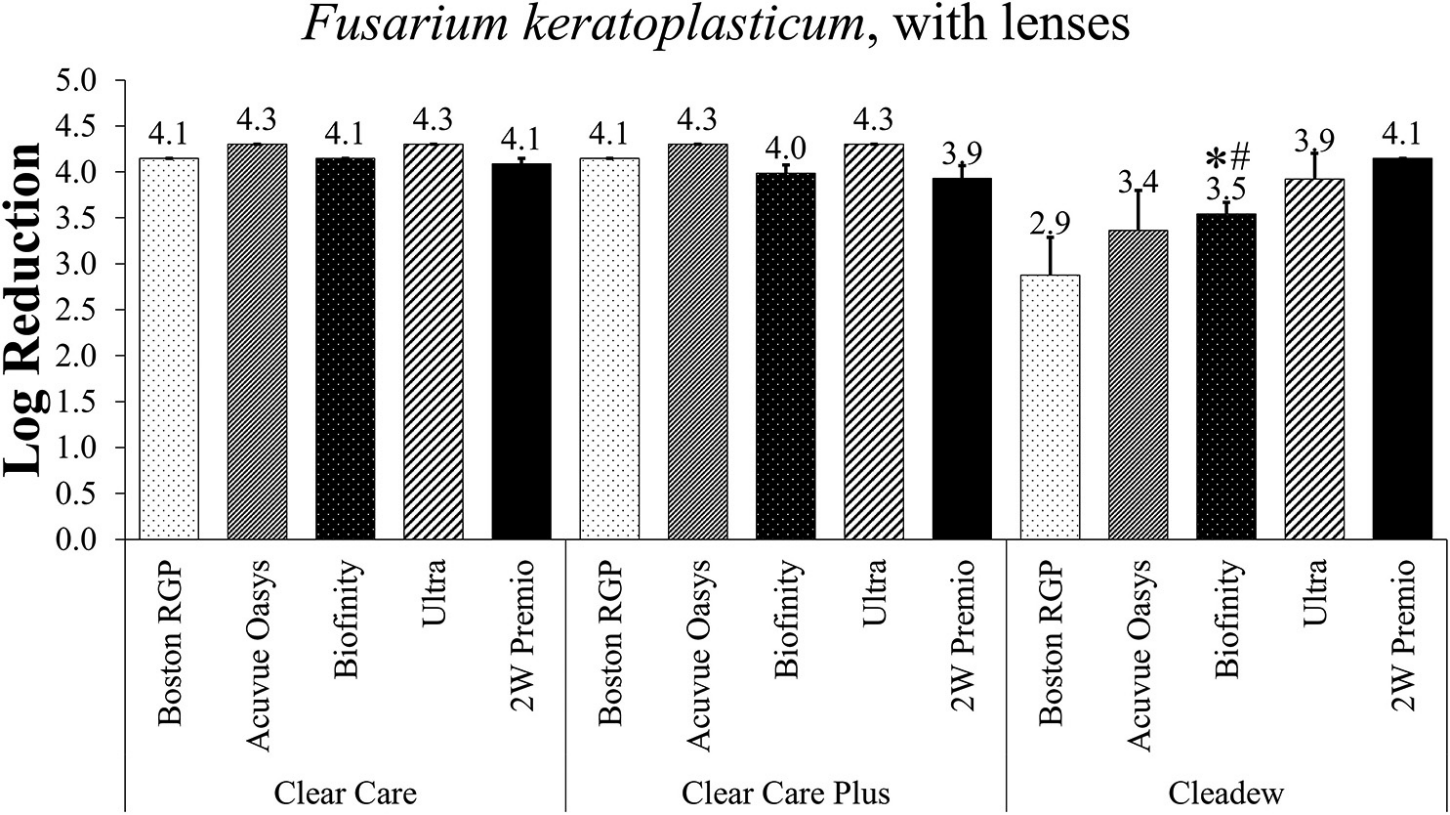
Disinfection efficacy of preservative-free disinfection systems against Fusarium keratoplasticum with contact lenses, at the manufacturer’s disinfection time. Disinfection efficacy is given as the mean ± standard error log reduction compared to the inoculum control. *, *P* < 0.05 versus Clear Care; #, *P* < 0.05 versus Clear Care Plus (one-way ANOVA; *n* = 3 to 6/group).

*F. keratoplasticum* can cause microbial keratitis and is a challenging microorganism which can produce differential results between products. Thus, *F. keratoplasticum* was then used to evaluate disinfection efficacy between disinfection solutions in combination with one of five contact lenses, Boston RGP, Acuvue Oasys, Biofinity, Ultra, or 2-week (2W) PremiO (Fig. 4). While Cleadew demonstrated the lowest log reduction averages, there were no significant differences between most of the products in combination with these lenses, after inoculation with *F. keratoplasticum*, due to the higher variation in the Cleadew tests. The only exception was for the Biofinity lenses, which produced a significantly lower log reduction of Fusarium when disinfected using Cleadew than with both Clear Care and Clear Care Plus.

**FIG 5:**
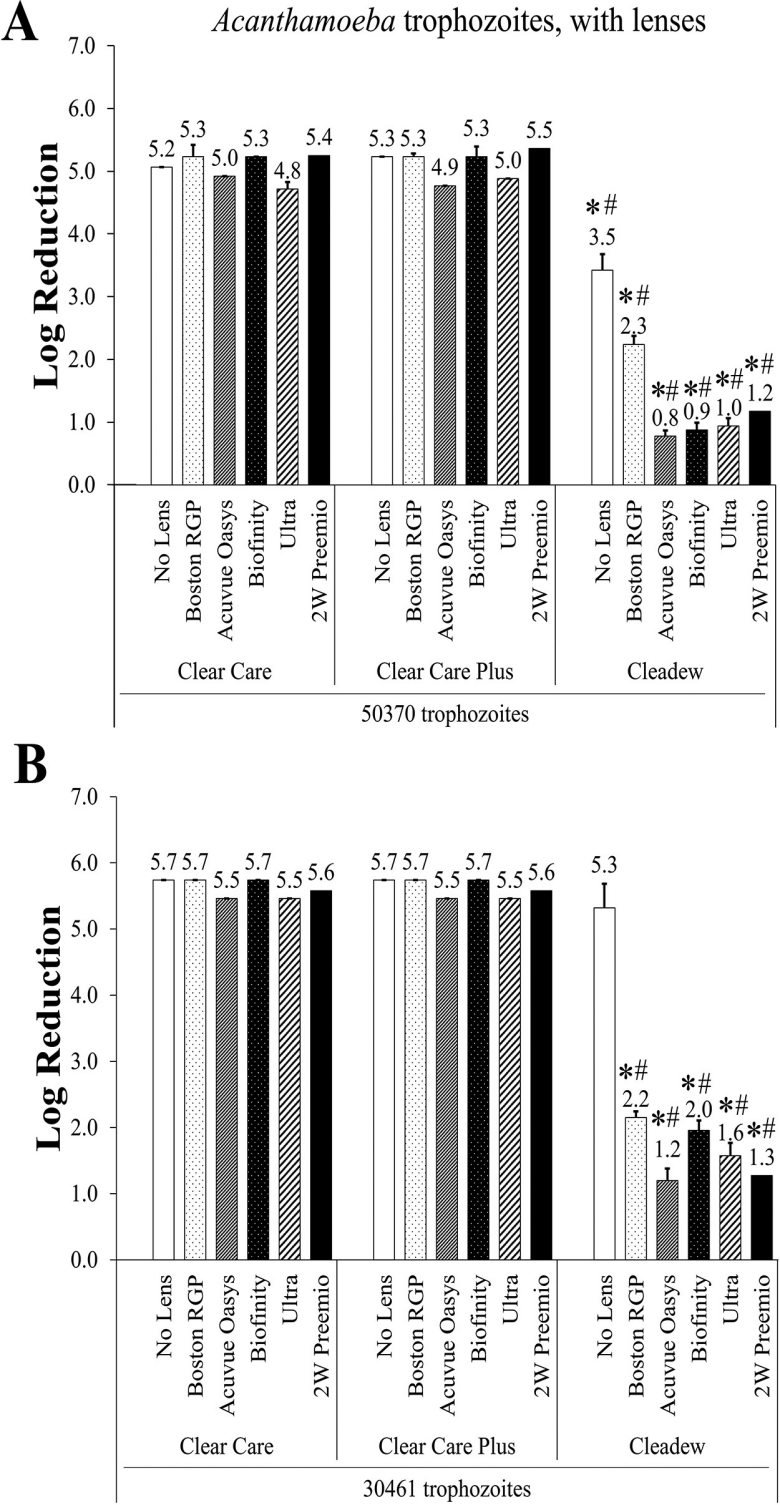
Disinfection efficacy of nonpreserved disinfecting solutions against *Acanthamoeba* trophozoites, with contact lenses, at the manufacturer’s disinfection time. (A) ATCC 50370 trophozoites; (B) ATCC 30461 trophozoites. Disinfection efficacy is given as the mean ± standard error log reduction compared to the inoculum control. *, *P* < 0.05 versus Clear Care; #, *P* < 0.05 versus Clear Care Plus (one-way ANOVA; *n* = 3 to 6/group).

These same lenses were also used to evaluate the disinfection efficacy of Clear Care, Clear Care Plus, and Cleadew against ATCC 30461 and ATCC 50370 *Acanthamoeba* trophozoites (Fig. 5). These strains were chosen for the with-lens challenges due to their extensive descriptions in antimicrobial efficacy literature, representation of the T4 genotype as it relates to *Acanthamoeba keratitis* infections, and scientific reproducibility (18). With the ATCC 30461 trophozoites, there were no differences in log reduction between disinfection products in the no-lens condition. Differences in log reduction between the ISO 14729 procedure and the no-lens controls for the ISO 18259 procedure are likely due to how each test is inoculated. There may be enhanced standard error in the no-lens ISO 18259 protocol due the lack of mixing prior to lens case closure. However, all lenses tested in this strain, i.e., Boston RGP, Acuvue Oasys, Biofinity, Ultra, and 2W PremiO, combined with Cleadew demonstrated significantly lower log reduction than when these lenses were combined with Clear Care or Clear Care Plus (*P* < 0.05). With the ATCC 50370 strain, when Cleadew was tested with the no-lens condition as well as all five lenses, this again produced significantly lower log reduction of *Acanthamoeba* trophozoites than either Clear Care or Clear Care Plus (*P* < 0.05). There were no differences between Clear Care and Clear Care Plus in the disinfection efficacy of *Acanthamoeba* trophozoites when tested in combination with contact lenses.

**FIG 6:**
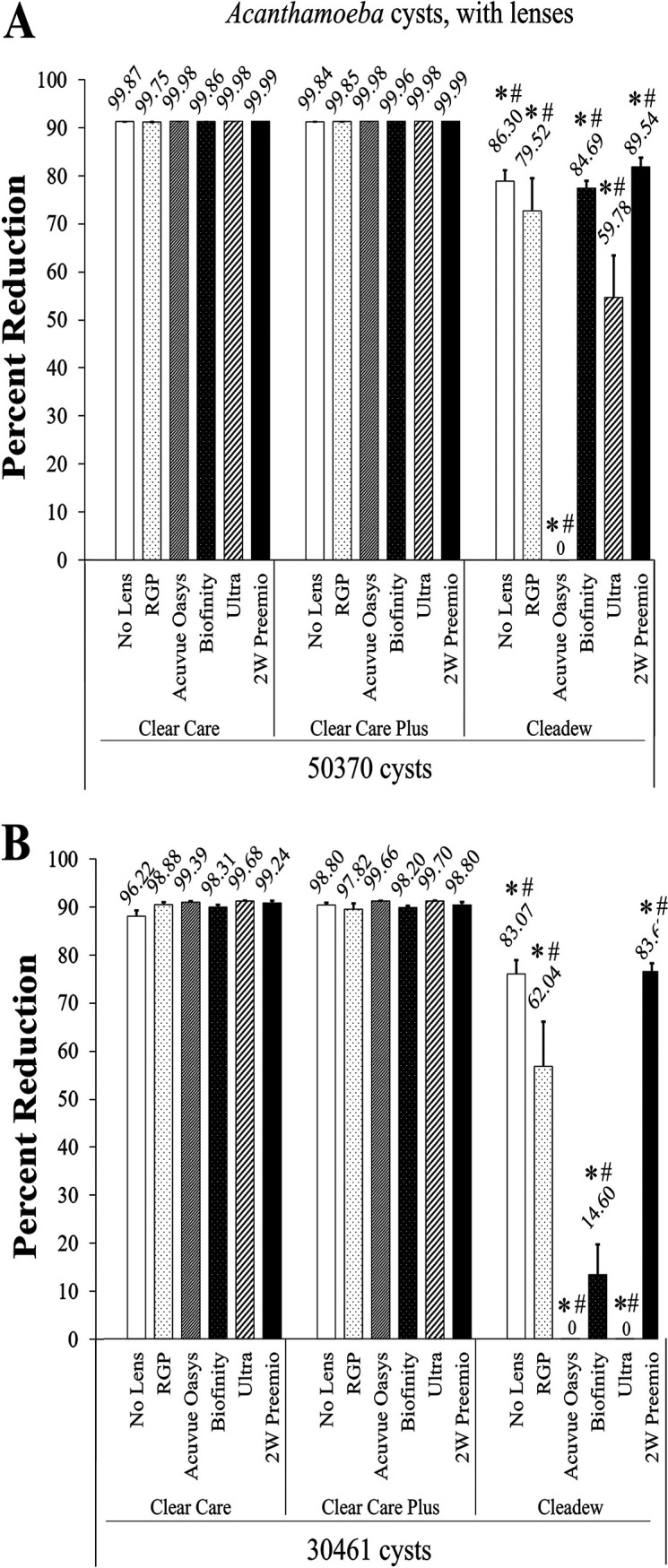
Disinfection efficacy of preservative-free disinfection systems against *Acanthamoeba* cysts, with contact lenses, at the manufacturer’s disinfection time. (A) ATCC 50370 cysts; (B) ATCC 30461 cysts. Disinfection efficacy is given as the mean ± standard error percent reduction compared to the inoculum control. *, *P* < 0.05 versus Clear Care; #, *P* < 0.05 versus Clear Care Plus (one-way ANOVA; *n* = 3 to 6/group).

Finally, the results of the most challenging disinfection trial, *Acanthamoeba* cysts combined with lenses, are presented in percent reduction to demonstrate the differentiation between products (Fig. 6). The results were highly similar between the ATCC 30461 and ATCC 50370 strains. With both strains, when Cleadew was combined with any of the five lenses, as well as the no-lens challenge, it produced significantly less disinfection efficacy than either Clear Care or Clear Care Plus when combined with these same lenses (*P* < 0.05).

## DISCUSSION

Microbial keratitis (MK) persists even in developed countries, largely due to improper contact lens care practices and/or ineffective contact lens care products (19–24). Within this, *Acanthamoeba* keratitis specifically remains as one of the most challenging ocular infections to treat, and *Acanthamoeba* cysts persist as an extremely resistant organism to biocidal activity (25, 26). However, all forms of keratitis present a significant threat to the eye, are difficult to diagnose, and may result in blindness (21). Therefore, it is imperative to understand which contact lens care products are able to eliminate common pathogens, with and without the presence of lenses. In the fight against MK, the preservative-free disinfection systems routinely demonstrate greater disinfection efficacy than the multipurpose solutions which rely on biocides and preservatives (11–13). The chief disinfecting agents in the preservative-free systems are either hydrogen peroxide or povidone-iodine. Thus, while there have been studies to understand the disinfection efficacy of these systems in general (11–13, 27–29), to our knowledge there has not been an investigation which directly compares the efficacies of these two specific preservative-free disinfection systems.

To standardize the measurement of the disinfection efficacy of contact lens care products across all market products, the ISO has published a uniform testing method for stand-alone (no contact lenses or cases) procedures, as well as the primary criteria for judging the disinfection efficacy results of these tests (16). The ISO 14729 applies to the microorganisms shown in Fig. 1, namely, S. aureus, P. aeruginosa, S. marcescens, C. albicans, and *F. keratoplasticum*. To meet these criteria, bacterial challenges must demonstrate at least a 3 log reduction, and fungal challenges must demonstrate at least a 1 log reduction at disinfection time. All three of the products tested—Clear Care, Clear Care Plus, and Cleadew—easily met and exceeded these primary criteria, and there were no significant differences between products in this test.

When a modified stand-alone procedure was applied to the trophozoites and cysts of four different *Acanthamoeba* strains, differences between both products and strains became evident. Of the trophozoites, only the ATCC 50370 strain did not produce differences between products, but the other three strains (ATCC 30461, ATCC 30868, and ATCC 50676) all resulted in Cleadew producing significantly less disinfection efficacy than the Clear Care products. All of the reductions for these products and strains against *Acanthamoeba* trophozoites were between 4.4 and 5.7 log, indicating pronounced disinfection efficacy. With the ATCC 50370 and ATCC 30461 strains, Clear Care and Clear Care Plus disinfection resulted in no survivors (<1 cell/mL). However, these same investigations against *Acanthamoeba* cysts produced a range of 0.5 to 3.0 log reduction, underscoring the relative difficulty of disinfecting any product from cysts, even when using extremely robust preservative-free disinfection systems. With the cyst experiments, the differentiation between products became more evident. Cleadew underperformed the Clear Care products in all four *Acanthamoeba* strains tested. Interestingly, different strains of cysts produced differing results between Clear Care products, indicating that cysts may produce results which are even more strain specific than those results found using trophozoites. ATCC 50370 and ATCC 30461 cysts produced no differences between Clear Care products, ATCC 30868 resulted in Clear Care having greater disinfection efficacy than Clear Care Plus, and ATCC 50676 resulted in Clear Care Plus having greater disinfection efficacy than Clear Care.

Following stand-alone examinations, representative microorganisms from each aforementioned group were used to examine the efficacy of the preservative-free disinfection systems in combination with contact lenses. These were performed in accordance with ISO 18259 to provide methodology for experiments using lenses (30). Similar to the stand-alone tests, there were minimal differences demonstrated between products when the lens-combined tests were used in conjunction with *F. keratoplasticum*. The log reduction values were largely similar between the stand-alone Fusarium tests and those with lenses. In the stand-alone tests, the Fusarium examinations resulted in a range of 4.1 to 4.3 log reduction between products, while in the tests with lenses, this log reduction range decreased to 2.9 to 4.3, although the lower results possessed larger error, resulting in no statistical differences. The only difference produced by Fusarium lens challenges were with the Biofinity lens, wherein the Cleadew disinfection efficacy was significantly lower than that of Clear Care or Clear Care Plus.

The *Acanthamoeba* ATCC 30461 and ATCC 50370 strains were used to perform the with-lens disinfection efficacy investigations between preservative-free disinfection systems. With the *Acanthamoeba* trophozoite tests, the Cleadew product almost universally underperformed the Clear Care products, except for the no-lens challenge with the ATCC 30461 strain. Except for the no-lens challenges, the Cleadew tests resulted in reductions between 0.8 and 2.3 log, while the Clear Care tests resulted in reductions of 4.8 to 5.7 log. Given these results, it is demonstrated that the Clear Care products maintained results similar to those of the stand-alone methodology (which produced a reduction of 4.7 to 5.7 log), while Cleadew was not able to achieve the same log reduction as it did during the stand-alone test (4.4 to 5.6 log). This indicates that the povidone-iodine disinfecting system may struggle to maintain disinfection efficacy when faced with the real-world challenge of contact lens disinfection, even with the relatively more susceptible trophozoite stage of *Acanthamoeba*. Additionally, there is a noted difference between the disinfection efficacies of stand-alone testing and no-lens testing. This is likely due to the slightly modified testing technique wherein the microorganism is added directly to the solution for a stand-alone test, versus the microorganism being added to the contact lens care case and then the disinfection solution is added afterwards for a no-lens test.

Finally, the preservative-free disinfection systems were challenged with ATCC 30461 and ATCC 50370 cysts in combination with contact lenses. This represents one of the most difficult disinfection challenges, as cysts are notoriously resistant to disinfection, and, as this study has noted, the addition of real-world with-lens challenges has the potential to reduce contact lens care solution disinfection efficacy. As some Cleadew results demonstrated <1 log reduction, this final value is presented in percent reduction in order to visualize efficacy when 90% reduction (1 log) is not achieved. In all challenges, Cleadew significantly underperformed the Clear Care products, and no differences in disinfection efficacy between the Clear Care products was demonstrated. The Clear Care products performed extremely similarly without lenses (2.0 to 3.0 log reduction) as with lenses (1.5 to 4.8 log reduction, or 96.22% to 99.99% reduction), across all challenges. Conversely, Cleadew struggled to match either its own stand-alone cyst results (0.5 to 1.4 log reduction) or the Clear Care products with-lens results, as the *Acanthamoeba* cyst Cleadew challenges resulted in a range of only 0% to 90% reduction across all challenges. Thus, these results indicate that with this study, only the hydrogen peroxide-based systems were able to maintain robust disinfection efficacy against *Acanthamoeba* cysts, with or without lenses present.

Notably, Clear Care and Clear Care Plus maintained their high antimicrobial efficacy in the presence and absence of contact lenses. As there is no risk of biocide uptake by contact lenses impacting efficacy as there is with multipurpose solutions (31), the lens case structure and microorganism adherence are major risks when adding contact lenses to disinfection efficacy studies. This study demonstrates that lenses had no impact on hydrogen peroxide products, indicating that Clear Care/Clear Care Plus lens case design (vertical baskets with platinum disc on the stem, suspended in the solution) did not affect efficacy, and Clear Care/Clear Care Plus was still effective even if microorganisms adhered to lenses. This is sharply contrasted with the results with Cleadew, which had previously shown high efficacy against *Acanthamoeba* trophozoites when tested without lenses. The addition of lenses caused a dramatic decrease in efficacy, possibly due to the lens case architecture. However, it is interesting to note that Cleadew’s loss of disinfection efficacy may be dependent on the composition of each lens, as well as on which microorganism is being used for the challenge, as we have previously shown that the disinfection efficacy of preserved contact lens care solutions can be significantly impacted by the presence of contact lenses and cases (31). For instance, 2W PremiO lenses maintain a high level of disinfection efficacy after challenge with Fusarium and *Acanthamoeba* cysts but not against *Acanthamoeba* trophozoites. The Cleadew lens case suspends contact lenses in baskets at the top of the solution, with limited proximity to the base of the case where the tablet sits and the povidone-iodine generation is occurring. The contact lenses are oriented concave up, which may prevent povidone-iodine from contacting the surface of the lens which has contact with the eye. Since a contact lens solution is always used in conjunction with contact lenses, the efficacy of a solution should always be assessed in their presence to ensure adequate disinfection for their intended use.

In conclusion, preservative-free disinfection systems, which rely on either hydrogen peroxide or povidone-iodine, have been marketed as potential resources for disinfection of contact lenses against all common pathogens which cause microbial keratitis. In particular, these products are hailed as some of the few disinfection systems which may be effective against both *Acanthamoeba* trophozoites and cysts. This study demonstrates for the first time that the hydrogen peroxide-based Clear Care products are significantly more efficacious than povidone-iodine-based systems against both *Acanthamoeba* trophozoites and cysts, particularly when challenges include contact lenses.

## MATERIALS AND METHODS

### Preparation of bacteria, yeast, and mold.

Organisms and methodology from the International Standard Organization (ISO) method 14729 were used (Table 1) (32). Bacterial cultures (S. aureus, P. aeruginosa, and S. marcescens) and yeast cultures (C. albicans) were incubated for 18 to 24 h at 30 to 35°C. Bacteria were incubated on soybean-casein digest agar, and yeast was incubated on Sabouraud dextrose agar. Cells were then harvested using sterile Dulbecco’s phosphate-buffered saline (DPBS). *F. keratoplasticum* cultures were incubated for 10 to 14 days at 20 to 25°C on potato dextrose agar (PDA). Spores were then harvested using sterile DPBS with 0.05% polysorbate 80. The suspensions were adjusted with sterile solution to concentrations of approximately 1.0 × 10^7^ to 1.0 × 10^8^ CFU per mL using a spectrophotometer set at a wavelength of 525 nm.

**TABLE 1:** Test organisms used, their species, isolation source, and whether or not they are required organisms under ISO 14729^*a*^

Microorganism	Species	Isolation source	ISO 14729 required organism
Staphylococcus aureus	ATCC 6538	Lesion	Required
Pseudomonas aeruginosa	ATCC 9027	Outer ear infection	Required
Serratia marcescens	ATCC 13880	Pond water	Required
Candida albicans	ATCC 10231	Man with bronchomycosis	Required
Fusarium keratoplasticum	ATCC 36031	Corneal ulcer	Required
Acanthamoeba castellanii	ATCC 50370	Eye infection	Not required
Acanthamoeba polyphaga	ATCC 30461	Corneal scrapings	Not required
Acanthamoeba castellanii	ATCC 30868	Cornea	Not required
Acanthamoeba mauritaniensis	ATCC 50676	Eye of human with *Acanthamoeba* keratitis	Not required

a All *Acanthamoeba* strains used were of the T4 genotype.

### Preparation of *Acanthamoeba*.

As previously described (33, 34), *Acanthamoeba* master culture plugs (Table 1) were used to inoculate T150 flasks containing 75 mL of axenic culture medium (AC6). Flasks were incubated for 3 days at 26 to 30°C. Trophozoites were then cultured in AC6 for an additional 3 days and evaluated for confluence. To create homogenous populations of cysts, trophozoites from AC6 flasks were transferred to nonnutrient agar plates and incubated for at least 7 days at 26 to 30°C. Following incubation, cysts were collected and washed with 0.5% SDS to lyse any immature cysts prior to use in testing. Inoculum suspensions were prepared by centrifugation at 500 rpm for 5 min at room temperature and pouring off the supernatant. The remaining amoeba pellet was then resuspended in 1/4× Ringer’s solution.

### Contact lens care solution antimicrobial activity procedure, without lenses.

In accordance with the ISO 14729 procedure, a 10-mL volume of Clear Care or Clear Care Plus (Table 2) was added to the manufacturer-provided lens cup. For Cleadew, an 8-mL volume of test sample was added to a manufacturer-provided lens cup, along with the disinfecting/neutralizing tablet. Microorganisms were then inoculated into the product with approximately 1% volume of the appropriate inoculum and thoroughly mixed. Lens cases were closed and stored at room temperature for the manufacturer’s recommended disinfection time.

**TABLE 2:** Products used, their manufacturers, and product details, including disinfection time and/or contact lens group^*a*^

Manufacturer	Product	Product details	DT or contact lens group
Alcon, Fort Worth, TX, USA	Clear Care	3% (wt/vol) hydrogen peroxide	6 h
	Clear Care Plus	3% (wt/vol) hydrogen peroxide	6 h
Ophtecs, Tokyo, Japan	Cleadew	Povidone-iodine (4 mg) and ascorbic acid (2 mg)	4 h
Johnson & Johnson, New Brunswick, NJ, USA	Acuvue Oasys	Senofilcon A	Group V
Bausch + Lomb, Rochester, NY, USA	Ultra	Samfilcon A	Group V
	Boston XO rigid gas-permeable lens	Fluorosilicone acrylate	Nonhydrogel group
CooperVision, Lake Forest, CA, USA	Biofinity	Comfilcon A	Group V
Menicon, Tokyo, Japan	2W PremiO	Mipafilcon A	Group V

a DT, disinfection time. See reference 36.

### Contact lens care solution procedure, with lenses.

In accordance with a modified ISO 18259 procedure for preservative-free products, examination of disinfection efficacy in the presence of contact lenses was performed. Prior to the addition of product to the lens case, in the “no-lens” condition, the bottom of the case was inoculated to contain approximately 1.0 × 10^5^ to 1.0 × 10^6^ CFU/mL. In the “with-lens” condition, this inoculum was applied directly to the lenses, which were placed in manufacturer-provided lens baskets. The inoculum was allowed to adhere for 3 min. Aliquots (8 to 10 mL) of the test solutions were added to the lens cups. The Cleadew tablet was then added to the Cleadew systems. Lens baskets were placed in the lens cup, and the lid was closed. At disinfection time, the lenses and total contents of lens care cases were transferred to a sterile tube and vortexed for 30 s. This solution was then evaluated for microbial load via serial dilution.

### Bacterial, yeast, and mold test quantification procedure.

Final concentrations of 1.0 × 10^5^ to 1.0 × 10^6^ CFU/mL were used for evaluation of test samples. One-milliliter aliquots of the inoculated test samples were removed at disinfection time, and serial dilutions were prepared. One milliliter of the test sample was added to 9.0 mL Dey-Engley (DE) broth, and serial dilutions were prepared. One-milliliter aliquots from each dilution were transferred to petri plates in duplicate. Twenty to 25 mL of Trypticase soy agar (TSA) containing 0.07% lecithin and 0.05% polysorbate 80 (TSA^++^) was poured for bacterial, yeast, and mold plates. Bacterial plates were incubated for a minimum of 2 days at 30 to 35°C, yeast plates were incubated for 3 to 5 days at 30 to 35°C, and mold plates were incubated for 5 to 7 days at 20 to 25°C. Following the incubation periods, plate counts were conducted and the numbers of organisms were recorded in CFU. CFU/mL was calculated based on the average for duplicate plates.

Inoculum controls were prepared for each microorganism. Sterile DPBS was used to prepare the bacterial and yeast controls, while sterile DPBS plus polysorbate 80 was used to prepare the mold controls. Each control was inoculated with an aliquot of a microorganism suspension containing approximately 1 × 10^7^ to 1 × 10^8^ CFU/mL to result in a final concentration of 1 × 10^5^ to 1 × 10^6^ CFU/mL. Inoculum controls were sampled immediately to determine the number of organisms inoculated into the test samples as well as to serve as the baseline for subsequent controls. Serial dilutions were prepared for each test control, and pour plates were incubated in the same manner as the test samples. The number of microorganisms (CFU/mL) was calculated based on the average count from duplicate plates.

Log reduction values were calculated by subtracting the log_10_ count of surviving organisms at a specified time from the log_10_ count of the initial organism inoculum control count.

### *Acanthamoeba* quantification procedure.

At the manufacturer’s disinfection time, 1 mL of the inoculated product was serially diluted in 1/4× Ringer’s solution. Each dilution was plated in quadruplicate on nonnutrient agar overlaid with a bacterial lawn. In addition to serial plating, trophozoite-inoculated samples of product were directly plated onto nonnutrient agar plates overlaid with bacterial lawn to lower the limit of quantification to <1 cell/mL.

For controls for each strain, 10 mL of 1/4× Ringer’s solution was placed in a sterile polystyrene screw-cap tube, and 0.1 mL of the appropriate amoeba inoculum was inoculated. One milliliter of the inoculated control was taken and serially diluted in 9-mL blanks containing 1/4× Ringer’s solution. Each dilution was plated in quadruplicate on nonnutrient agar overlaid with a bacterial lawn. All inoculated controls were performed in triplicate. Cyst and trophozoite plates were both incubated at 26 to 30°C for at least 14 days. Each test control was inoculated with an aliquot (0.1 mL) of an organism suspension containing approximately 1.0 × 10^6^ to 1.0 × 10^8^ CFU/mL to result in a final concentration of 1.0 × 10^4^ to 1.0 × 10^6^ CFU/mL depending on the type of *Acanthamoeba*.

Plates were inspected with an inverted microscope through day 14 using ×4 magnification for the presence of viable amoebae as indicated by proliferation of amoebae on the bacterial lawns. Results were recorded for each well, and survivors were quantified using the Reed and Muench computation (35). Log reduction values were calculated by subtracting the log_10_ count of surviving microorganisms at a specified time from the log_10_ count of the initial microorganism test control count.

### Statistics.

All comparisons were examined via one-way analysis of variance (ANOVA) followed by Tukey’s test *post hoc* analysis. Significance was set at a *P* of <0.05.
